# Patients’ preferences for osteoporosis drug treatment: a discrete-choice experiment

**DOI:** 10.1186/ar4465

**Published:** 2014-01-31

**Authors:** Mickaël Hiligsmann, Benedict G Dellaert, Carmen D Dirksen, Trudy van der Weijden, Stefan Goemaere, Jean-Yves Reginster, Verity Watson, Annelies Boonen

**Affiliations:** 1 Department of Health Services Research, School for Public Health and Primary Care (CAPHRI), Maastricht University, P.O. Box 616, 6200 MD Maastricht, the Netherlands; 2 Department of Business Economics, Erasmus Rotterdam University, Rotterdam, the Netherlands; 3 Department of Clinical Epidemiology and Medical Technology Assessment, CAPHRI, Maastricht University, Maastricht, the Netherlands; 4 Department of Family Medicine, CAPHRI, Maastricht University, Maastricht, the Netherlands; 5 Department of Rheumatology and Endocrinology, Ghent University Hospital, Gent, Belgium; 6 Department of Public Health, Epidemiology and Health Economics, University of Liege, Liege, Belgium; 7 Health Economics Research Unit, Institute of Applied Health Sciences, University of Aberdeen, Aberdeen, UK; 8 Department of Internal Medicine, CAPHRI, Maastricht University, Maastricht, the Netherlands

## Abstract

**Introduction:**

The patient’s perspective is becoming increasingly important in clinical and policy decisions. In this study, we aimed to evaluate the preferences of patients with, or at risk of, osteoporosis for medication attributes, and to establish how patients trade between these attributes.

**Methods:**

A discrete choice experiment survey was designed and patients were asked to choose between two hypothetical unlabelled drug treatments (and an opt-out option) that vary in five attributes: efficacy in reducing the risk of fracture, type of potential common side-effects, mode and frequency of administration and out-of-pocket costs. An efficient experimental design was used to construct the treatment option choice sets and a mixed logit panel data model was used to estimate patients’ preferences and trade-offs between attributes.

**Results:**

A total of 257 patients with, or at risk of, osteoporosis completed the experiment. As expected, patients preferred treatment with higher effectiveness and lower cost. They also preferred either an oral monthly tablet or 6-month subcutaneous injection above weekly oral tablets, 3-month subcutaneous, 3-month intravenous or yearly intravenous injections. Patients disliked being at risk of gastro-intestinal disorders more than being at risk of skin reactions and flu-like symptoms. There was significant variation in preferences across the sample for all attributes except subcutaneous injection.

**Conclusions:**

This study revealed that osteoporotic patients preferred 6-month subcutaneous injection and oral monthly tablet, and disliked gastro-intestinal disorders. Moreover, patients were willing to pay a personal contribution or to trade treatment efficacy for better levels of other attributes. Preferences for treatment attributes varied across patients and this highlights the importance of clinical decision-making taking individual preferences into account to improve osteoporosis care.

## Introduction

The patient’s perspective is becoming increasingly important in clinical and policy decisions. Information about what patients need and prefer, and how they value various aspects of a health intervention can be useful when designing and evaluating healthcare programs [1]. A better understanding of patients’ preferences for treatment can help health professionals to improve disease management. When differences in efficacy or safety do not determine the choice of a specific treatment, patient’s satisfaction with therapy is important [2]. Addressing patients’ concerns with treatment and involving them in clinical decision-making may also improve adherence [1]. Patients increasingly want to be informed by their doctors, and to be active in clinical decision-making [3,4]. In recent years, discrete choice experiments (DCEs) have been increasingly used to elicit patients’ preferences for healthcare [5,6]. DCEs can quantify the relative importance of the various attributes that characterize a treatment and allow the trade-offs that respondents make between these to be quantified [7].

The aim of this study was to evaluate osteoporotic patients’ preferences for medication attributes using a DCE, and to establish how patients make trade-offs between these attributes. This study differs from previously published DCEs in osteoporosis in several ways [8-10]. First, this study includes recently introduced routes and timing of administration (for example, subcutaneous and intravenous injection) and the nature of potential side-effects. Given potential differences in preferences between administration schemes, information on patients’ preferences for these new administration schemes would be extremely useful for health professionals and decision-makers [11]. Second, this study expands the population studied to include men. Third, a rigorous qualitative research was performed to select medication attributes [12].

## Methods

### Discrete choice experiment

A DCE describes an intervention by its attributes (for example, effectiveness, side-effects, costs) and reports how patient’s preference for an intervention are influenced by the type and levels of these attributes [7]. In the DCE, patients were asked to choose between two unlabelled drug treatments (A and B) and a no treatment (opt-out) option. The alternative treatments varied in several attributes, and patients were asked to select the treatment they would prefer. Patients were asked to make a series of such hypothetical choices. This research followed published DCEs guidelines [1,13] and used rigorous methods to select treatment attributes, to design the DCE and to conduct the statistical analysis.

### Attributes and levels

The identification and selection of the DCE attributes is fundamental to obtaining valid results [14,15]. We conducted a nominal group technique to select the DCE attributes [14]. Full details on this are provided elsewhere [12]. In brief, patients’ group discussions (four to eight participants per group, *n*_total_ = 26) were conducted to prioritize a list of potentially important attributes of osteoporosis drug treatment. This list was developed from a literature review and discussions with experts. A ranking exercise and group discussions revealed five attributes that were consistently identified as important for patients: effectiveness, side-effects, mode and frequency of administration and out-of-pocket cost (Table 1) [12]. Levels were assigned to these attributes based on the current treatment using a literature review and discussion with experts (*n* = 5). For the side-effects of treatment, we focused on the types of common side-effects [16].

**Table 1:** Attributes and levels for osteoporosis drug treatment

	
Efficacy in reducing the risk of future fractures	20%
	30%
	40%
	50%
Possible side effects (affecting one in 50 patients)	Gastrointestinal disorders
	Flu-like symptoms
	Skin reactions
Mode of administration	Oral tablet
	Subcutaneous injection
	Intravenous injection
Frequency of administration	Weekly
	Monthly
	Every 3 months
	Every 6 months
	Yearly
Cost to you (per month)	€5
	€15
	€25
	€40
	€60

### Experimental design

**Figure 1:**
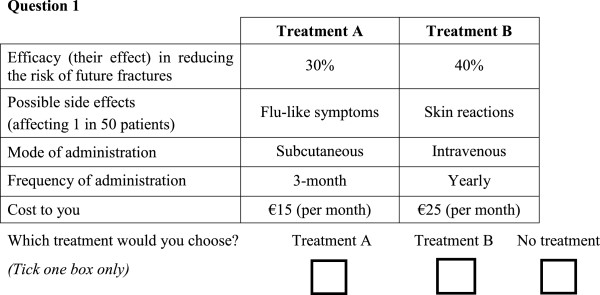
Example of a choice set.

It is not feasible to present an individual with all possible treatment combinations from the attributes and levels in Table 1. Experimental design techniques were used to draw a subset of treatment profiles to present to respondents in the DCE [5]. Specifically, a Bayesian efficient experimental design was used to select the subset using Ngene software (version 1.1.1) [17] to select the subset. This experimental design maximizes the precision of estimated parameters (by maximizing the *D* efficiency – a summary measure of the variance covariance matrix) for a given number of choice questions [18]. In this study, 15 choice sets were created. An example of a choice set is shown in Figure 1.

The construction of an efficient experimental design depends on patients’ preferences, so we conducted a pilot DCE study (*n* = 10). We used the pilot results to obtain preliminary information about patients’ preferences and then used this information to create the experimental design for the main study. The pilot DCE experimental design used *a priori* information about patients’ preferences based on literature review [9] and discussions during the qualitative research (for example, higher effectiveness is preferred). We also wished to avoid presenting respondents with implausible treatment options (for example, a yearly oral tablet), and therefore we restricted the experimental design to include only realistic combinations between mode and frequency of administration that could appear in the design (that is, oral weekly or monthly tablets, subcutaneous injection every 3 or 6 months, and intravenous injection every 3 months or yearly). The experimental design based on pilot preference information suggested that 200 respondents would be sufficient power to detect the significance of most parameters.

### Questionnaire, data collection and patient recruitment

In the questionnaire, patients received a thorough description of the DCE task. The attributes and levels were carefully explained and an example of a completed choice set was provided. One of the choice questions was asked twice to assess test–retest reliability. Each patient therefore received 16 choice sets. After completion of the choice tasks, respondents were asked how difficult they found the choice tasks on a seven-point scale. The DCE task is presented in Additional file 1. The questionnaire also asked questions on patients’ characteristics. Individual 10-year probabilities of a hip fracture and a major osteoporotic fracture (FRAX score) [19] were calculated for each respondent by a doctor/researcher and added to the questionnaire afterwards.

The questionnaire was developed in English by a working group that included a patient and clinical and DCE experts, and was approved by two native English speakers, experts in osteoporosis. The questionnaire was then translated into French and Dutch by a medical translation company specializing in the translation of patient reported outcome measures (Pharma Quest Ltd, Oxford, United Kingdom) and the translation was checked and approved by two native French and Dutch speakers with medical backgrounds. The questionnaire was pilot tested with 15 patients (French-speaking *n* = 10, Dutch-speaking *n* = 5) to check interpretation problems and face validity; no wording problems arose and only minor changes to layout were made.

Consecutive patients with, or at risk of, osteoporosis to whom medication (or lifestyle changes) was at least proposed were recruited during outpatients’ clinics in two Belgian osteoporosis centers (Ghent and Liège). Explanation of the task and an example choice task was provided by the doctor or a researcher. The questionnaire was mainly completed by the patient at home and returned in a postage-paid envelope. Very few patients completed the questionnaire at the clinic but without any assistance from the doctor/researcher. Approval for this study was obtained from the ethics committee of Maastricht University Medical Center who coordinated this project and participants gave informed written consent.

### Statistical analyses

From the DCE, we observe the respondent’s choice of one treatment from the three alternatives presented in each choice set. Responses are analyzed based on random utility theory [20]. In this case, the utility that a patient *i* assigns to a treatment *j*, *V*_*ij*_, is modeled as the sum of two parts: a systematic part based on the attributes included in the DCE, and an error part *ϵ*_*ijt*_. We specify *V*_*ij*_ as:

$$\begin{array}{ll} V_{\mathrm{ij}}= & \beta_{0}+\left(\beta_{1}+\eta_{1i}\right){\;\text{EFFICACY}}_{j}+\left(\beta_{2}+\eta_{2i}\right){\;\text{COST}}_{j}\\ & +\left(\beta_{3}+\eta_{3i}\right)\;{\text{ORAL}}_{1}M_{j}+\left(\beta_{4}+\eta_{4i}\right)\;{\text{SUB}}_{3}M_{j}\\ & +\left(\beta_{5}+\eta_{5i}\right)\;{\text{SUB}}_{6}M_{j}+\left(\beta_{6}+\eta_{6i}\right)\;{\text{INT}}_{3}M_{j}\\ & +\left(\beta_{7}+\eta_{7i}\right)\;{\text{INT}}_{1}Y_{j}+\left(\beta_{8}+\eta_{8i}\right){\;\text{FLUSYMPT}}_{j}\\ & +\left(\beta_{9}+\eta_{9i}\right){\;\text{SKINREACT}}_{j}+\epsilon_{\mathrm{ij}} \end{array}$$

where β_0_ is the constant reflecting the preferences for selecting treatment relative to no treatment, β_1_ to β_9_ are the mean attribute utility weights in the population, and η_1i_ to η_9i_ are error terms capturing individual-specific unexplained variation in the utility weights. Dummy coding was used (for ease of interpretation of the results) to describe all categorical variables (β_3_ to β_9_). Reference levels for mode of administration and for side effects are weekly oral tablet and risk of gastrointestinal disorders, respectively. The sign of the coefficient reflects whether the attribute/level has a positive or a negative effect on treatment utility compared with the base level. The value of a coefficient indicates the relative importance of the attribute/level.

When developing a statistical model of respondents’ choice it is important to account for respondents completing up to 15 choice tasks each and to allow preferences for treatment to vary across the sample. A mixed logit panel data model was therefore estimated using Nlogit, version 5 [21,22]. This model allows model parameters (preferences) to vary in the population. This variation is achieved by specifying a random parameter that has a distribution and estimating the mean (β) and standard deviation of the error term (η) to capture the parameter’s distribution. If the standard deviation is significantly different from zero, this is interpreted as evidence of significant preference variation for the attribute in the sample.

Initially, we estimated models in which preferences for all attributes could vary in the population, and then in the final model – those attributes for which the estimated standard deviation was not significant (5% level) – the preferences were specified to be the same in the population (fixed parameters). The random parameters for cost and efficacy were drawn from a log-normal distribution – this allows us to constrain the parameter estimate to be either negative (for cost) or positive (for efficacy) [22]. All other random parameters were drawn from a normal distribution. The estimation was conducted using 2,000 Halton draws.

We also calculated marginal willingness to pay (WTP) and marginal willingness to trade efficacy (WTTE) of the attributes/levels. This allows us to compare preferences for all attributes measured with a common and interpretable metric either money or efficacy. A WTP (or WTTE) value represents how much one is willing to pay (or to trade) for a one-unit change in the attribute, and is calculating by taking the ratio of the mean parameter for the attribute/level to the mean parameter related to the cost (or efficacy). As the cost and efficacy variables were estimated as random parameters, the WTP and WTTE calculations must take this into account. As recommended in this case, the conditional constrained parameters were used [22].

The mixed logit model identifies attributes for which there is significant preference variation, but it does not explain why this variation exists. To understand the potential sources of preference variation, additional analyses included covariates (such as gender, age) in the model one by one. Significant covariates were then included together and nonsignificant covariates were excluded from this model. An adjusted pseudo-*R*^2^ and finite Akaike information criterion were used to enable comparison of models with and without covariates. We also tested whether patients using a specific mode of administration had a stronger preference for this administration scheme by incorporating interactions between levels and covariates. Furthermore, to explore the impact of respondents who failed the test–retest, a sensitivity analysis was conducted by excluding these individuals. A subgroup analysis was also conducted in patients with high risk of fractures (defined as a FRAX major risk >10%) and in patients with low risk of fractures (defined as a FRAX major risk ≤10%). To assess the significance of the differences between populations, a joint model was estimated using interaction terms.

## Results

### Patients’ characteristics

A total of 301 questionnaires were distributed to patients; 268 were returned, representing a response rate of 89%. Eleven questionnaires were excluded because the patient did not complete at least five choice sets in the DCE task. A total of 257 (85%) questionnaires were included for data analysis. Respondents’ sociodemographics and health characteristics are presented in Table 2. There was no restriction on participation based on patients’ race and ethnicity but patients were mainly Caucasian.

**Table 2:** Patients’ characteristics

	
Age (years, mean ± standard deviation)	67.1 ± 10.4
Female gender	83.3%
Educational level	
Primary	8.4%
Some high school	35.9%
High school graduate	30.3%
College or university	25.5%
Size of household	
One person	29.9%
Two people	55.1%
Three or more people	15.0%
Monthly household income	
Up to €999	5.5%
€1,000 to 1,499	33.1%
€1,500 to 1,999	19.1%
€2,000 to 2,499	17.8%
€2,500 to 2,999	11.9%
€3,000+	12.7%
Diagnosis of osteoporosis	89.8%
Years since osteoporosis (mean ± standard deviation)	8.9 ± 0.3
With prior fracture(s)	52.5%
In the last year	22.8%
Patients on osteoporotic treatment	69.8%
Administration mode of current treatment	
Oral	72.2%
Subcutaneous injection	15.4%
Intravenous injection	12.4%
Number of co-treatments	
Zero or one	19.3%
Two or three	40.6%
Four or more	40.2%
10-year probability of a major osteoporotic fracture (FRAX) (mean ± standard deviation)	14.3 ± 7.5%
10-year probability of a hip fracture (FRAX) (mean ± standard deviation)	6.1 ± 5.3%

The difficulty of the task on a seven-point scale (1 = extremely easy to 7 = extremely difficult) was estimated on average between 3 and 4. The task was found to be extremely easy for 35 patients (13.6%) while 19 patients (7.4%) gave a score of 6 of 7. A total of 219 patients (85.2%) chose the same alternative in the test–retest exercise. This is in line with existing test–retest results [15].

### Patients’ preferences

The distribution of choices across the choice sets is presented in Additional file 2. The main results of the mixed logit model are presented in Table 3. The estimated coefficients for efficacy and costs had the expected sign and were statistically significant. The positive sign of the efficacy parameter indicates that respondents prefer higher treatment efficacy, and the negative sign of the cost parameter indicates that respondents prefer paying less money for treatment. Patients prefer a 6-month subcutaneous injection and a monthly oral tablet compared with a weekly oral tablet (base level). There were no significant differences between weekly oral tablet, 3-monthly subcutaneous injection and yearly intravenous treatment; and no significant differences between 6-monthly subcutaneous injection and monthly oral tablet. Regardless of the administration mode, patients preferred a longer dosing regimen (monthly vs. weekly oral tablet; 6-monthly vs. 3-monthly subcutaneous injection; yearly vs. 3-monthly intravenous treatment). The positive sign for the two side-effect parameters indicates that patients disliked being at risk of gastrointestinal disorders (base) more than being at risk of skin reactions or flu-like symptoms.

**Table 3:** Results from the panel mixed logit model

**Attributes and levels**	**Estimate (95% confidence interval)**	***P *** **value**	**Standard deviation**
Constant	0.90*** (0.62 to 1.17)	0.00	–
Efficacy (1% risk reduction)	0.07*** (0.05 to 0.08)^a^	0.00	1.19*** (1.06 to 1.30)
Cost per month (€1)	-0.05*** (–0.04 to –0.06)^a^	0.00	1.24*** (1.09 to 1.39)
Drug administration (reference level: weekly oral tablet)			
Monthly oral tablet	0.69*** (0.36 to 1.03)	0.00	0.92*** (0.65 to 1.19)
Subcutaneous injection 3-monthly	0.16 (–0.09 to 0.42)	0.21	NS^b^
Subcutaneous injection 6-monthly	0.75*** (0.44 to 1.07)	0.00	NS
Intravenous injection 3-monthly	-0.57** (–1.12 to –0.01)	0.05	2.62*** (2.04 to 3.20)
Intravenous injection yearly	0.28 (–0.12 to 0.68)	0.17	1.56*** (1.17 to 1.94)
Side-effects (reference level: gastrointestinal disorders)			
Flu-like symptoms	0.97*** (0.76 to 1.18)	0.00	0.90*** (0.65 to 1.15)
Skin reactions	0.63*** (0.41 to 0.85)	0.00	1.04*** (0.81 to 1.26)

Number of observations 3,822 (257 respondents × 15 choices, minus 33 missing values).

Pseudo-*R*^2^ = 0.42; log-likelihood = –2,456.03; Akaike information criterion = 1.29.

^a^For the coefficients of efficacy and cost to you, exp(β) is shown. The standard deviation of the log-normal distribution is reported. ^b^Not significant and not included in the final model. **P < 0.05. ***P < 0.01.

The standard deviation parameters were statistically significant for all attributes except for the subcutaneous injection, suggesting the presence of preference variation in the importance of the attribute/level across respondents. To gain more insight into how preferences vary, the distributions of the parameters or kernel density estimates of the individual parameter are presented in Additional file 3.

### Willingness to pay

The WTP and WTTE for attributes/levels are presented in Table 4. For example, respondents were willing to pay a personal contribution of €19.53 more per month or to give up 13.52% of a drug’s efficacy for the treatment mode of 6-month subcutaneous injection rather than a weekly oral tablet.

**Table 4:** **Willingness to pay and willingness to trade efficacy for osteoporosis medication attributes**^**a**^

**Attributes and levels**	**Willingness to pay**	**Willingness to trade efficacy**
	**(€ per month)**	**(% risk reduction)**
Efficacy (1% risk reduction)	3.73 (3.01 to 4.44)	–
Cost (€1)	–	-2.27 (–1.58 to –2.96)
Drug administration (reference level: weekly oral tablet)		
Monthly oral tablet	16.16 (12.85 to 19.47)	-10.16 (–7.88 to –12.50)
Subcutaneous injection 3-monthly	4.24 (3.72 to 4.76)	-2.93 (–2.57 to –3.30)
Subcutaneous injection 6-monthly	19.53 (17.15 to 21.92)	-13.52 (–11.82 to –15.22)
Intravenous injection 3-monthly	-15.28 (–23.23 to –7.34)	8.66 (14.31 to 3.01)
Intravenous injection yearly	11.75 (5.64 to 17.85)	-5.83 (–1.88 to –9.77)
Side-effects (reference level: gastrointestinal disorders)		
Flu-like symptoms	25.21 (13.06 to 20.50)	-16.68 (–14.20 to –19.16)
Skin reactions	16.78 (13.06 to 20.50)	-9.48 (–7.13 to –11.83)

Data presented as mean (95% confidence interval). A positive willingness to pay means that patients are willing to pay a personal contribution for the attribute/level, while a negative willingness to trade efficacy means that patients are willing to give up treatment efficacy for the attribute/level. ^a^Using the conditional constrained distribution.

### High-risk patients versus low-risk patients

The results of the model for high-risk and low-risk patients are presented in Table 5. Significant differences in preferences were found between these patient groups for the effectiveness and cost of treatment – the interactions between risk group and effectiveness and cost parameters were significant (5% level)). Lower effectiveness and higher costs are more acceptable for patients with high-risk of fractures. In addition, high-risk patients attached a higher (negative) value to being at risk for skin reactions than low-risk patients, and the constant (that is, preferences for drug treatment *per se*) was higher for high-risk patients. Preferences for drug administration did not differ significantly between patient groups.

**Table 5:** Differences between high-risk and low-risk patients’ preferences for osteoporosis drug treatment

**Attributes and levels**	**High risk patients**	**Low-risk patients**	***P *** **value**^**a**^
	**(FRAX major >10%)**	**(FRAX major ≤10%)**	
Number of patients	139	114	
Pseudo-*R*^2^	0.39	0.42	
Log-likelihood	-1,378.35	-1,085.55	
Constant	1.50*** (1.17 to 1.83)	-0.05 (–0.52 to 0.43)	0.01
Efficacy (1% risk reduction)	0.04*** (0.03 to 0.04)	0.14*** (0.11 to 0.17)	0.00
	SD: 1.65***	SD: 1.01***	
Cost per month (€1)	-0.02*** (–0.02 to –0.03)	-0.08*** (–0.06 to –0.09)	0.00
	SD: 1.45***	SD: 0.67***	
Drug administration (reference level: weekly oral tablet)			
Monthly oral tablet	0.57** (0.08 to 1.06)	1.14*** (0.47 to 1.82)	0.14
	SD: 0.94***	SD: 1.87***	
Subcutaneous injection 3-monthly	0.14 (–0.19 to 0.47)	0.28 (–0.17 to 0.74)	0.14
	SD: NS	SD: NS	
Subcutaneous injection 6-monthly	0.57*** (0.17 to 0.96)	1.55*** (0.97 to 2.14)	0.06
	SD: NS	SD: NS	
Intravenous injection 3-monthly	-0.28 (–0.88 to 0.31)	-0.24 (–1.39 to 0.91)	0.25
	SD: 1.82***	SD: 4.84***	
Intravenous injection yearly	0.28 (–0.13 to 0.69)	0.75** (0.05 to 1.45)	0.33
	SD: 0.81***	SD: 2.15***	
Side effects (reference level: gastrointestinal disorders)			
Flu-like symptoms	0.66*** (0.36 to 0.95)	1.51*** (1.07 to 1.95)	0.57
	SD: 0.91***	SD: 1.18***	
Skin reactions	0.45** (0.05 to 0.85)	0.49** (0.10 to 0.87)	0.05
	SD: 1.31***	SD: 1.04***	

Data presented as estimate (95% confidence interval). SD, standard deviation; NS, not significant. ^*a*^*P* value was estimated in a joint model with interaction terms. **P < 0.05. ***P < 0.01.

### Additional analyses

Excluding respondents who failed the test–retest (*n* = 38) had no impact on the relative importance of the attributes (see Additional file 3). The inclusion of more covariates into the model did not significantly improve the adjusted McFadden’s pseudo-*R*^2^ but reduced the sample size by 17% due to missing values (see Additional file 3). We therefore did not include these covariates in the reference model. The only significant covariate effects we observed were that the preference for drug treatment was higher for men and patients with higher income (monthly household income > €2,500 per month). Other parameters were not affected by the inclusion of covariates. In addition, patients did not significantly prefer their current mode of administration over another mode of administration.

## Discussion

This study suggests that patients with, or at risk of, osteoporosis have preferences for medications’ attributes and are willing to trade between attributes when making treatment choices. Our results are consistent with *a priori* expectations that patients prefer higher efficacy, lower costs and less frequent dosing regimens. In addition, patients preferred 6-month subcutaneous injection or monthly oral tablet over weekly oral tablet or intravenous injections, and they disliked being at risk for gastrointestinal disorders. Patients are willing to trade efficacy or to pay a personal contribution for better levels of other attributes. For most of the attributes, there was significant variation in patients’ preferences.

Previous DCEs have investigated women’s preference for osteoporosis drug treatment [8-10]. Our results confirm the findings of de Bekker-Grob and colleagues that patients prefer monthly oral tablet to weekly oral tablet [9] and those of Darba and colleagues suggesting no significant difference in preference between weekly oral regimen and yearly intravenous injection [8]. Fraenkel and colleagues also showed that preferences are strongly influenced by the route of administration but suggest a majority (65%) of Americans preferred yearly intravenous infusion over weekly oral tablet [10]. Our study expands on the insights of these studies. We expand the population studied to include men, new recent administration routes and frequencies (for example, 6-month subcutaneous injection) and the nature of potential side-effects. A rigorous qualitative research was also conducted to select attributes.

Results of this study could be very useful for health professionals and decision-makers, especially given the poor adherence to weekly oral regimens and the potential differences in healthcare costs associated with osteoporosis medications. Nonadherence to medication is a major problem among patients with osteoporosis and affects considerably the effectiveness and cost-effectiveness of drug therapy [23,24]. Determinants of poor adherence include inconvenient regimens [25]. In our study, many patients preferred a 6-monthly subcutaneous injection compared with weekly oral tablets and yearly intravenous injections. The recent introduction of 6-monthly subcutaneous injection of denosumab [26] and the recognition of the importance of patients’ preferences could therefore potentially improve patient satisfaction and adherence with therapy [27]. Our results could also inform healthcare decision-making, in particular for drug reimbursement, where insights into the preferences of patients groups should be taken into account alongside medical and economic considerations [28].

In addition, the variation in the patients’ preferences for attribute levels observed in our study highlights the importance to take into account individual preferences into clinical decision-making to improve osteoporosis care. Relying solely on sample average preferences will probably be insufficient to optimize medical doctors’ sensitivity to the preferences of an individual and unique patient during a consultation. Informing individual patients about alternative options and their outcomes, and involving them in decision-making, would be very important to improve patient satisfaction and the outcome of medical care [29].

Our study has some potential limitations. First, although consecutive patients were invited to participate in this study, we cannot exclude selection bias as some patients did not want, or were not able, to fill in the questionnaire. Second, generalizability and transferability of our findings may be limited by recruiting patients from two osteoporosis centers in one country only. A cross-country comparison is ongoing in seven European countries. Preferences for attributes/levels may differ according to a number of factors including age, income, education or prior fractures [30]. While we do not find evidence of preference variation associated with these factors in our study, the cross-country comparison will investigate this further. Third, we focused on the nature of common side-effects, not on their frequency and rare complications. Rare adverse events will be as (in)frequent in all categories of anti-resorptive drugs. Therefore, adding osteonecrosis of the jaw and atypical femoral fracture to the side-effect attribute would probably not differentiate between patient preferences across existing drugs. Attributes were selected using a rigorous qualitative method as recommended in good practice guidelines [1,13]. Finally, one could point out that the individual 10-year probability of fractures was not provided to the patients before completing the questionnaire. Only 35 (14%) patients reported knowing their FRAX score.

## Conclusions

This study revealed that osteoporotic patients prefer 6-monthly subcutaneous injection and oral monthly tablets, and disliked gastrointestinal disorders. Moreover, they were willing to trade efficacy or to pay a personal contribution for their preferred outcomes. We found differences in preferences across patients, which highlights the importance of clinical decision-making taking individual preferences into account to improve osteoporosis care.

## Abbreviations

DCE: discrete choice experiment; WTP: willingness to pay; WTTE: willingness to trade efficacy.

## Competing interests

MH has received research grants and/or lecture fees from Amgen, Novartis, Pfizer, Servier and SMB. SG has received speakers fees and/or research support from Amgen, Daiichi-Sankyo, Eli Lilly, Glaxo SmithKline, Merck Sharp & Dohme, Novartis, Nycomed, Warner-Chillcott, Sanofi-Aventis, Servier, and Roche. J-YR received consulting fees, paid advisory boards, lecture fees, and/or grant support from Servier, Novartis, Negma, Lilly, Wyeth, Amgen, GlaxoSmithKline, Roche, Merckle, Nycomed, NPS, Theramex, UCB, Merck Sharp and Dohme, Rottapharm, IBSA, Genevrier, Teijin, Teva, Ebewee Pharma, Zodiac, Analis, Novo-Nordisk, and Bristol Myers Squibb. The remaining authors state that they have no competing interests.

## Authors’ contributions

MH, AB, BGD, CDD, TvdW and VW were responsible for design of the study. SG and J-YR were responsible for data collection. MH, AB, BGD, CDD and VW were responsible for data analysis. MH had full access to the data and takes responsibility for the integrity of the data and the accuracy of the data analysis. MH drafted the manuscript and all other authors revised it critically for important intellectual content. All authors approved the final version.

## Supplementary Material

Additional file 1 The questionnaire given to the patients. Click here for file

Additional file 2 A table presenting the distribution of choices (treatment A, treatment B, no treatment) across the 15 choice sets. Click here for file

Additional file 3 Shows tables and figures presenting additional results including estimates of models with excluding respondents who failed the test–retest or with inclusion of covariates, and kernel density estimates of the individual parameters. Click here for file
