# Supplementary material for: Patients’ preferences for osteoporosis drug treatment: a discrete-choice experiment
Source: Arthritis Res Ther. 2014 Jan 31;16(1):R36. doi: 10.1186/ar4465 (PMC3979104; doi:10.1186/ar4465)
Supplement: Additional file 1 — The questionnaire given to the patients. [file ar4465-S1.pdf]

Additional file 1: questionnaire

Task: Making choices for drug therapy

In this task, we are interested in your opinion on drug therapy for osteoporosis. In order to identify your opinion, we would like to ask you to make a series of choices between different drug therapies.

To help you make these choices, please read carefully through the following information.

<u>The task</u>

Please imagine that this is your first visit to a rheumatology clinic. You have recently been diagnosed with osteoporosis and your doctor has advised you that you should start taking medication.

In each of the following 16 choices, you will be offered two drug therapies (**A** or **B**). In each choice please state whether you would choose to take drug therapy A, drug therapy B, or **no treatment**. If you choose no treatment you would not receive treatment for your osteoporosis (and please assume that there are no other available treatment options).

The drug therapies you will be offered will differ in five ways: (1) their effect in reducing the risk of fractures, (2) side-effects, (3) mode of administration, (4) frequency of administration and (5) out-of-pocket costs. These 5 characteristics of the drug therapies will now be explained.

- **<u>Efficacy (their effect) in reducing the risk of relevant fractures</u> (**such as fractures of hip, wrist, shoulder or vertebrae) – this may be ***20%, 30%, 40%*** or ***50%***.

  Percentages can be a little difficult to understand in this context; so to help explain please refer to the following example:
  Based on individual risk factors (such as age, sex, weight, family history of fractures, previous fractures), assume that a person's risk for having a fracture in the next 10 years is 20%. Assume this value represents the average risk of fractures in elderly osteoporotic women.
  In that case, it would mean that:
  - Without any treatment, 20 women out of 100 will sustain a fracture within the next 10 years (20%)
  - With a treatment efficacy of 50%, 10 out of 100 women will sustain a fracture
  - With a treatment efficacy of 40%, 12 out of 100 women will sustain a fracture
  - With a treatment efficacy of 30%, 14 out of 100 women will sustain a fracture
  - With a treatment efficacy of 20%, 16 out of 100 women will sustain a fracture

- **Side-effects** – these may be ***gastro-intestinal disorders*** (such as nausea, diarrhea, constipation, vomiting, and loss of appetite), ***skin reactions*** (such as mild redness possibly itching followed by some roughness and feeling of tightness) and ***flu-like symptoms*** (low grade fever, mild muscle and headache).
  Assume **only one in every 50** patients treated will have a side effect. Each of these side-effects is relatively mild, disappears after a few days and has no long-term or severe consequences.
  It is important to remember that the frequency of the occurrence of any side effects during the treatment is NOT dependent on the frequency of the administration of the drug.
- **Mode of administration** – this may be ***oral tablet, subcutaneous, intravenous***
  - ***Oral tablet***: This would be taken in the morning, at least 30 minutes before breakfast and it is important not to lie down for at least an hour after taking the tablet
  - ***Subcutaneous (injection under the skin)***: injection under your skin given to you by a doctor or nurse (at home or at the physician's office)
  - ***Intravenous (injection into the vein)***: given by infusion into your vein in a clinic or hospital setting. The infusion usually takes approximately 15 minutes
- **Frequency of administration** – this may be ***weekly, monthly, once every 3 months, once every 6 months*** or ***annually***
- **Personal contribution (cost to you) per month** – This may be ***€5, €15, €25, €40*** *or* ***€60***. If you are currently a medical card holder and therefore not paying for your drugs, for the purpose of this questionnaire can you please imagine that you should pay this amount yourself every month

Example of the task (please do not fill in)

	**Treatment A**	**Treatment B**
Efficacy in reducing the risk of future fractures	30%	20%
Possible side effects (affecting 1 in 50 patients)	Gastro-intestinal side effects	Flu-like symptoms
Mode of administration	Intravenous	Oral tablet
Frequency of administration	Once yearly	Once weekly
Cost to you	€40 (per month)	€25 (per month)

Which treatment would you choose?	Treatment A	Treatment B	No treatment
(Tick one box only)	☐	X	☐

In this example, you are asked to choose between **treatment A** which reduces your risk of future fractures by 30%, has some gastro-intestinal side-effects in one in 50 patients, is administered once per year by an intravenous infusion (into a vein) and has an out-of-pocket

cost to you of €40 per month; **Treatment B** which reduces your risk of future fracture by 20%, can give mild flu-like symptoms to 1 in 50 patients, is taken as an oral tablet once weekly and the costs to you would be €25 per month; and **no treatment**. In the example above, the patient chooses treatment B, and therefore ticks the box treatment B.

Please choose from each of the following 16 choice sets your treatment of choice for the management of osteoporosis.

Question 1

	**Treatment A**	**Treatment B**
Efficacy (their effect) in reducing the risk of future fractures	30%	40%
Possible side effects (affecting 1 in 50 patients)	Flu-like symptoms	Skin reactions
Mode of administration	Subcutaneous	Intravenous
Frequency of administration	3-month	Yearly
Cost to you	€15 (per month)	€25 (per month)

Which treatment would you choose?	Treatment A	Treatment B	No treatment
(Tick one box only)	☐	☐	☐

Question 2

	**Treatment A**	**Treatment B**
Efficacy (their effect) in reducing the risk of future fractures	20%	50%
Possible side effects (affecting 1 in 50 patients)	Flu-like symptoms	Skin reactions
Mode of administration	Intravenous	Oral tablet
Frequency of administration	Yearly	Weekly
Cost to you	€60 (per month)	€5 (per month)

Which treatment would you choose?	Treatment A	Treatment B	No treatment
(Tick one box only)	☐	☐	☐

Question 3

	Treatment A	Treatment B
Efficacy (their effect) in reducing the risk of future fractures	20%	40%
Possible side effects (affecting 1 in 50 patients)	Skin reactions	Flu-like symptoms
Mode of administration	Subcutaneous	Intravenous
Frequency of administration	3-month	3-month
Cost to you	€5 (per month)	€60 (per month)

Which treatment would you choose? Treatment A ☐ Treatment B ☐ No treatment ☐

(Tick one box only)

Question 4

	Treatment A	Treatment B
Efficacy (their effect) in reducing the risk of future fractures	50%	30%
Possible side effects (affecting 1 in 50 patients)	Skin reactions	Flu-like symptoms
Mode of administration	Subcutaneous	Oral tablet
Frequency of administration	6-month	Monthly
Cost to you	€25 (per month)	€25 (per month)

Which treatment would you choose? Treatment A ☐ Treatment B ☐ No treatment ☐

(Tick one box only)

Question 5

	Treatment A	Treatment B
Efficacy (their effect) in reducing the risk of future fractures	40%	30%
Possible side effects (affecting 1 in 50 patients)	Skin reactions	Gastro-intestinal disorders
Mode of administration	Subcutaneous	Subcutaneous
Frequency of administration	6-month	3-month
Cost to you	€60 (per month)	€5 (per month)

Which treatment would you choose? Treatment A ☐ Treatment B ☐ No treatment ☐

(Tick one box only)

Question 6

	Treatment A	Treatment B
Efficacy (their effect) in reducing the risk of future fractures	30%	20%
Possible side effects (affecting 1 in 50 patients)	Gastro-intestinal disorders	Flu-like symptoms
Mode of administration	Oral tablet	Oral tablet
Frequency of administration	Weekly	Monthly
Cost to you	€60 (per month)	€15 (per month)

Which treatment would you choose? Treatment A ☐ Treatment B ☐ No treatment ☐

(Tick one box only)

Question 7

	Treatment A	Treatment B
Efficacy (their effect) in reducing the risk of future fractures	30%	40%
Possible side effects (affecting 1 in 50 patients)	Gastro-intestinal disorders	Skin reactions
Mode of administration	Oral tablet	Subcutaneous
Frequency of administration	Weekly	3-month
Cost to you	€5 (per month)	€60 (per month)

Which treatment would you choose? Treatment A ☐ Treatment B ☐ No treatment ☐

(Tick one box only)

Question 8

	Treatment A	Treatment B
Efficacy (their effect) in reducing the risk of future fractures	50%	20%
Possible side effects (affecting 1 in 50 patients)	Skin reactions	Gastro-intestinal disorders
Mode of administration	Subcutaneous	Subcutaneous
Frequency of administration	3-month	6-month
Cost to you	€40 (per month)	€5 (per month)

Which treatment would you choose? Treatment A ☐ Treatment B ☐ No treatment ☐

(Tick one box only)

Question 9

	Treatment A	Treatment B
Efficacy (their effect) in reducing the risk of future fractures	40%	30%
Possible side effects (affecting 1 in 50 patients)	Flu-like symptoms	Gastro-intestinal disorders
Mode of administration	Intravenous	Subcutaneous
Frequency of administration	3-month	6-month
Cost to you	€15 (per month)	€40 (per month)

Which treatment would you choose?	Treatment A	Treatment B	No treatment
(Tick one box only)	☐	☐	☐

Question 10

	Treatment A	Treatment B
Efficacy (their effect) in reducing the risk of future fractures	30%	40%
Possible side effects (affecting 1 in 50 patients)	Skin reactions	Gastro-intestinal disorders
Mode of administration	Oral tablet	Subcutaneous
Frequency of administration	Monthly	3-month
Cost to you	€5 (per month)	€60 (per month)

Which treatment would you choose?	Treatment A	Treatment B	No treatment
(Tick one box only)	☐	☐	☐

Question 11

	Treatment A	Treatment B
Efficacy (their effect) in reducing the risk of future fractures	50%	20%
Possible side effects (affecting 1 in 50 patients)	Gastro-intestinal disorders	Skin reactions
Mode of administration	Oral tablet	Intravenous
Frequency of administration	Monthly	Yearly
Cost to you	€40 (per month)	€15 (per month)

Which treatment would you choose?	Treatment A	Treatment B	No treatment
(Tick one box only)	☐	☐	☐

Question 12

	Treatment A	Treatment B
Efficacy (their effect) in reducing the risk of future fractures	20%	50%
Possible side effects (affecting 1 in 50 patients)	Flu-like symptoms	Gastro-intestinal disorders
Mode of administration	Subcutaneous	Oral tablet
Frequency of administration	6-month	Monthly
Cost to you	€15 (per month)	€40 (per month)

Which treatment would you choose? Treatment A ☐ Treatment B ☐ No treatment ☐

(Tick one box only)

Question 13

	Treatment A	Treatment B
Efficacy (their effect) in reducing the risk of future fractures	40%	20%
Possible side effects (affecting 1 in 50 patients)	Flu-like symptoms	Skin reactions
Mode of administration	Intravenous	Intravenous
Frequency of administration	Yearly	3-month
Cost to you	€40 (per month)	€25 (per month)

Which treatment would you choose? Treatment A ☐ Treatment B ☐ No treatment ☐

(Tick one box only)

Question 14

	Treatment A	Treatment B
Efficacy (their effect) in reducing the risk of future fractures	20%	50%
Possible side effects (affecting 1 in 50 patients)	Gastro-intestinal disorders	Flu-like symptoms
Mode of administration	Intravenous	Subcutaneous
Frequency of administration	3-month	6-month
Cost to you	€25 (per month)	€40 (per month)

Which treatment would you choose? Treatment A ☐ Treatment B ☐ No treatment ☐

(Tick one box only)

Question 15

	Treatment A	Treatment B
Efficacy (their effect) in reducing the risk of future fractures	40%	30%
Possible side effects (affecting 1 in 50 patients)	Gastro-intestinal disorders	Flu-like symptoms
Mode of administration	Oral tablet	Oral tablet
Frequency of administration	Monthly	Weekly
Cost to you	€25 (per month)	€15 (per month)

Which treatment would you choose? Treatment A ☐ Treatment B ☐ No treatment ☐

(Tick one box only)

Question 16

	Treatment A	Treatment B
Efficacy (their effect) in reducing the risk of future fractures	50%	20%
Possible side effects (affecting 1 in 50 patients)	Skin reactions	Gastro-intestinal disorders
Mode of administration	Subcutaneous	Subcutaneous
Frequency of administration	3-month	6-month
Cost to you	€40 (per month)	€5 (per month)

Which treatment would you choose? Treatment A ☐ Treatment B ☐ No treatment ☐

(Tick one box only)

Could you please state, on the following scale of 1 to 7, how easy or difficult this first task has been for you (i.e. the 16 choice questions). *(Circle one number only)*

Extremely easy						Extremely difficult
1	2	3	4	5	6	7
