# Supplementary material for: Patients’ preferences for osteoporosis drug treatment: a discrete-choice experiment
Source: Arthritis Res Ther. 2014 Jan 31;16(1):R36. doi: 10.1186/ar4465 (PMC3979104; doi:10.1186/ar4465)
Supplement: Additional file 2 — A table presenting the distribution of choices (treatment A, treatment B, no treatment) across the 15 choice sets. [file ar4465-S2.pdf]

Additional file 2 - Distribution of choices across the choice sets

	Treatment A	Treatment B	No treatment	Missing
Question 1	108	126	19	4
Question 2	25	215	15	2
Question 3	148	78	30	1
Question 4	179	63	14	1
Question 5	138	90	29	0
Question 6	37	194	26	0
Question 7	147	82	26	2
Question 8	139	88	28	2
Question 9	164	60	30	3
Question 10	186	46	23	2
Question 11	139	83	32	3
Question 12	112	119	23	3
Question 13	144	60	48	5
Question 14	47	177	30	3
Question 15	125	111	19	2
