# Supplementary material for: Patients’ preferences for osteoporosis drug treatment: a discrete-choice experiment
Source: Arthritis Res Ther. 2014 Jan 31;16(1):R36. doi: 10.1186/ar4465 (PMC3979104; doi:10.1186/ar4465)

Additional file 3 - Additional results

Model 1. Panel mixed logit model including only patients who were reliable in the test-retest exercise

Attributes and levels	Estimate (95% CI)	P Value	Standard deviation
Constant	1.02*** (0.74 to 1.31)	0.00	---
Efficacy (1% risk reduction)	0.05*** (0.04 to 0.07)	0.00	1.20*** (1.00 to 1.39)
Cost per month (€1)	-0.04*** (-0.04 to -0.05)	0.00	1.16*** (1.02 to 1.31)
Drug administration (reference level: weekly oral tablet)			
Monthly oral tablet	0.58** (0.22 to 0.95)	0.00	0.95*** (0.67 to 1.22)
Subcutaneous 3-month	0.11 (-0.16 to 0.38)	0.44	NS
Subcutaneous 6-month	0.63*** (0.29 to 0.98)	0.00	NS
Intravenous 3-month	-0.45 (-1.07 to 0.17)	0.15	2.60*** (1.94 to 3.26)
Intravenous yearly	0.09 (-0.36 to 0.53)	0.70	1.58*** (1.13 to 2.03)
Side effects (reference level: gastro-intestinal disorders)			
Flu-like symptoms	0.94*** (0.70 to 1.17)	0.00	0.90** (0.64 to 1.17)
Skin reactions	0.61*** (0.37 to 0.85)	0.00	1.00** (0.76 to 1.23)

Number of observations 3252 (219 X 15 choices, minus 33 missing values)

Pseudo R-squared = 0.41; Log-likelihood = -2092.78; AIC = 1.298.

* p<0.10, ** p<0.05, *** p<0.01; NS Not significant

Model 2. Panel mixed logit model including covariates

Attributes and levels	Estimate (95% CI)	P Value	Standard deviation
Constant	0.77*** (0.45 to 1.09)	0.00	---
Efficacy (1% risk reduction)	0.05*** (0.05 to 0.06)	0.00	1.23*** (1.13 to 1.33)
Cost per month (€1)	0.05*** (0.04 to 0.06)	0.00	1.17*** (1.03 to 1.31)
Men	0.84*** (0.48 to 1.20)	0.00	---
High income	0.28* (-0.04 to 0.59)	0.09	---
Drug administration (reference level: weekly oral tablet)			
Monthly oral tablet	0.67*** (0.28 to 1.06)	0.00	1.03*** (0.69 to 1.36)
Subcutaneous 3-month	0.06 (-0.24 to 0.35)	0.55	NS
Subcutaneous 6-month	0.65*** (0.31 to 1.00)	0.00	NS
Intravenous 3-month	-0.66** (-1.27 to -0.04)	0.04	2.96*** (2.25 to 3.68)
Intravenous yearly	0.17 (-0.30 to 0.64)	0.48	1.79*** (1.30 to 2.27)
Side effects (reference level: gastro-intestinal disorders)			
Flu-like symptoms	1.12*** (0.86 to 1.37)	0.00	0.99*** (0.70 to 1.27)
Skin reactions	0.57*** (0.34 to 0.81)	0.00	0.99*** (0.72 to 1.26)

Number of observations = 3215 (216 respondents X 15 choices, minus 25 missing values)

Pseudo R-squared = 0.42; Log-likelihood = -2062.73; AIC =1.29

* p<0.10, ** p<0.05, *** p<0.01; † NS Not significant

Other covariates including age, education level, prior fractures, diagnosis of osteoporosis, size of household, being on treatment and number of co-treatments were not significant.

Figure 1. Kernel density for the random parameters

The kernel densities provide a visual representation of the possible variation in preferences for each of the attributes. The graphs indicate that there is significant variation in preferences for all attributes. For the mode of administration attribute levels, there is preference variation across each of the modes. However, the variation differs by mode. While there is preference variation for a monthly oral tablet compared with a weekly oral tablet, the majority of respondents prefer a monthly oral tablet as indicated by the majority of the distribution of the preferences being positive. However, for intravenous three monthly intravenous injection, the distribution of the preferences is both positive and negative indicating that while some patients significantly preferred this mode others did not.

A. Efficacy

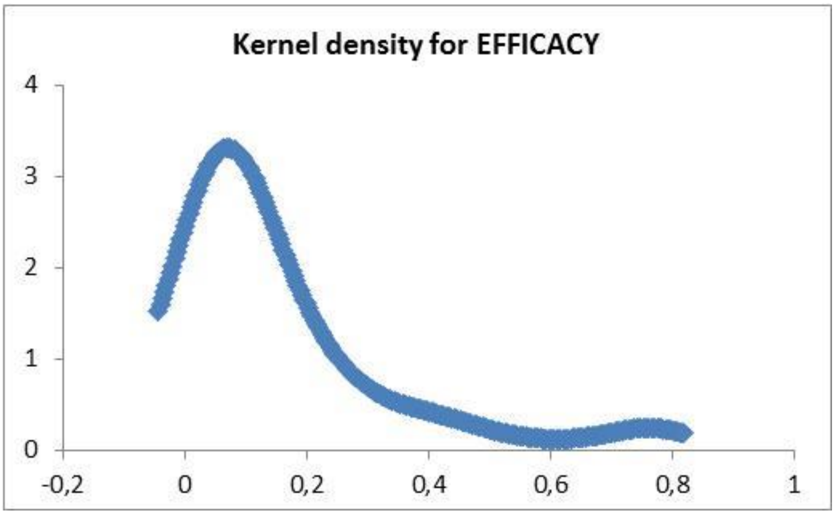

B. <u>Cost</u>

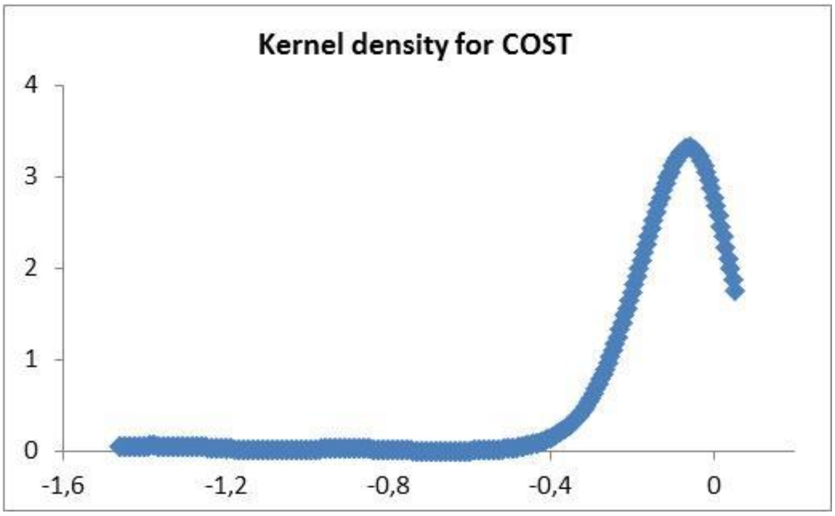

C. <u>Oral monthly tablet</u>

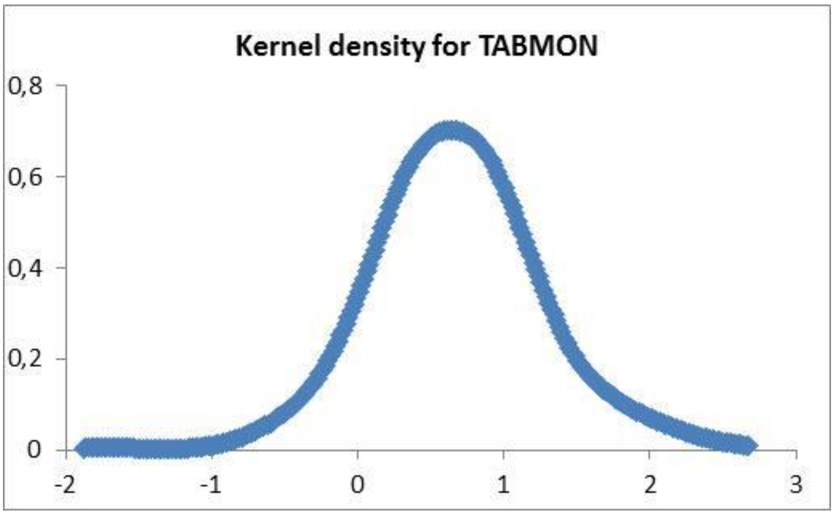

D. Intravenous every 3 months

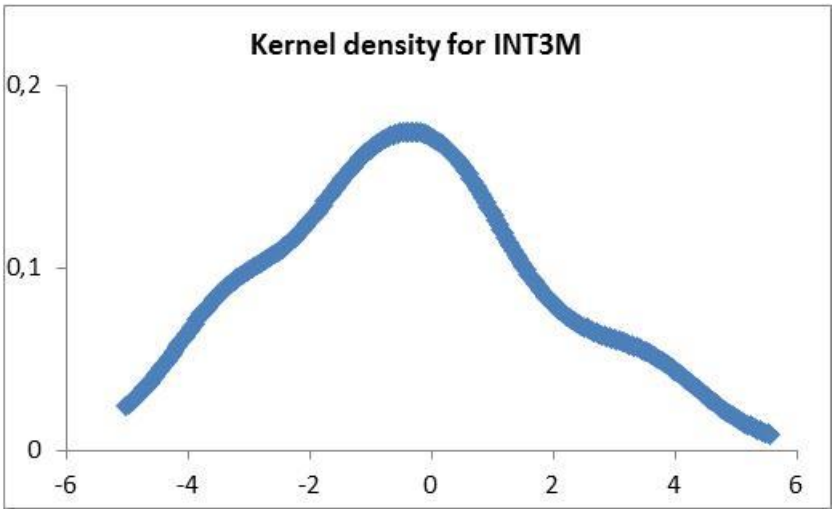

E. Yearly intravenous

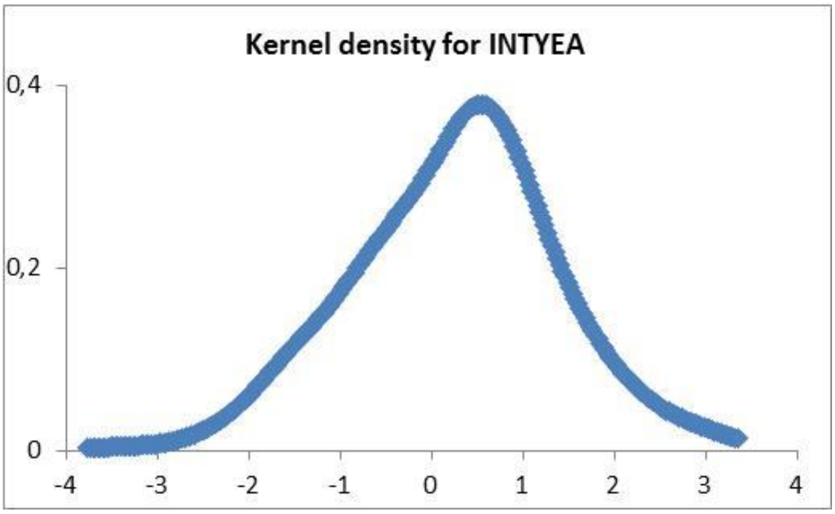

F. Skin reactions

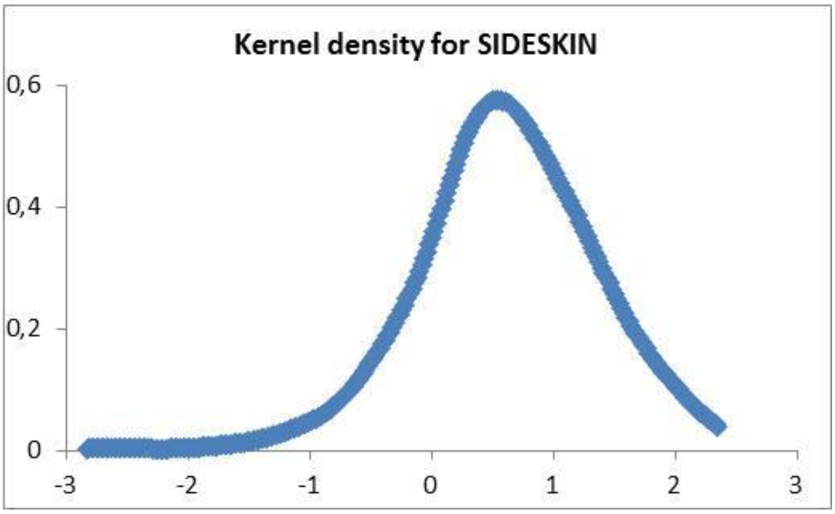

G. Flu-like symptoms

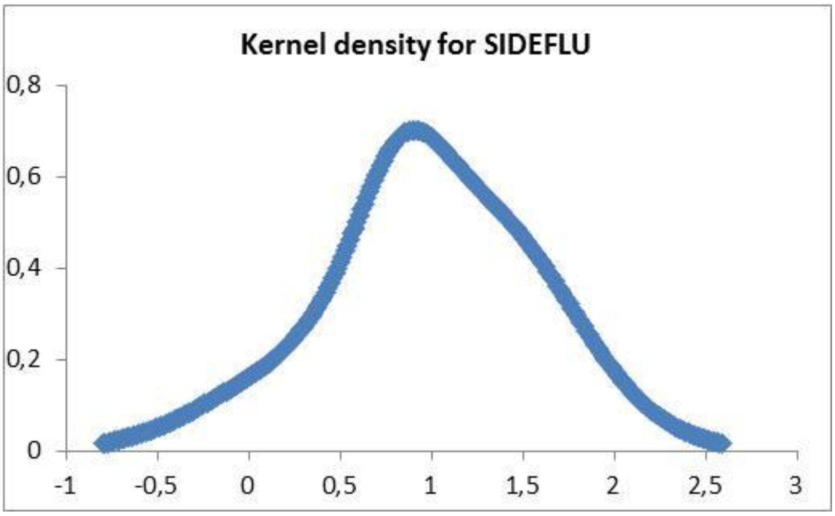

Supplement: Additional file 3 — Shows tables and figures presenting additional results including estimates of models with excluding respondents who failed the test–retest or with inclusion of covariates, and kernel density estimates of the individual parameters. [file ar4465-S3.pdf]
